# Mediation analysis for integrating motor symptoms of Parkinson’s disease, cognitive function, and gait characteristics

**DOI:** 10.3389/fneur.2025.1659581

**Published:** 2025-10-13

**Authors:** Wenchao Yin, Hong Gao, Ruichen Liu, Chenxin Shen, Yue Liu, Cui Wang

**Affiliations:** ^1^Department of Neurology, Central Hospital of Dalian University of Technology, Dalian, China; ^2^Department of Physics, Dalian Maritime University, Dalian, China; ^3^Shenyang Fire Research Institute of M.E.M, Shenyang, China; ^4^Department of Neurology, The Fifth People’s Hospital of Shenyang, Shenyang, China

**Keywords:** Parkinson’s disease, motor symptom, cognitive function, gait characteristic, mediation effect

## Abstract

**Background:**

In Parkinson’s disease (PD) patients, the severity of motor symptoms is closely related to the degree of gait disorder and cognitive impairment; notably, the latter also exhibits a significant correlation with gait disorder. Evidently, there exists a complex relationship between motor symptoms, cognitive function, and gait characteristics.

**Methods:**

This study aims to conduct an in-depth analysis of the relationships among MDS-UPDRS III score, MoCA score, and gait parameters by constructing a mediation model.

**Results:**

We found that a higher MDS-UPDRS III score was associated with a smaller plantar dorsiflexion angle, slower velocity, and worse swing phase symmetry, and these associations were not mediated by the MoCA score. Age was a confounder in the relationship between MDS-UPDRS III score, MoCA, and velocity. A higher MDS-UPDRS III score was associated with shorter stride length, and this association was partially mediated by the MoCA score.

**Conclusion:**

The results indicated that, when analyzing plantar dorsiflexion angle and swing phase symmetry in PD patients, the influence of motor symptoms was dominant; when analyzing stride length, motor symptoms and cognitive function need to be considered simultaneously; when analyzing velocity, the influence of motor symptoms and age should be focused on.

## 1 Introduction

The motor symptoms of Parkinson’s disease (PD) are complex and diverse, with gait disorder being one of the important clinical manifestations (1). Typical motor symptoms such as bradykinesia and rigidity directly affect the normal gait pattern (2). The gait characteristics of PD usually include reduced walking speed, shortened step length, and gait instability (2). Moreover, with the progression of the disease, these gait problems will gradually worsen (3) and severely affect the patients’ daily activities and the quality of life (4). Therefore, an accurate, scientific, timely, and reliable gait monitoring system is of crucial significance for clinicians to achieve accurate diagnosis, optimize treatment plans, and effectively monitor the progression of the disease.

Based on these, research studies on the quantitative assessment of gait have achieved profound progress. Numerous studies analyzed the characteristics of gait disorder in PD patients by comparing them with healthy controls (5, 6). Some researchers focused on exploring gait characteristics in early-stage (7) and drug-naive patients (8). In addition, researchers also attempted to explore gait characteristics under different Hoehn and Yahr (H&Y) stages (9) and section III of Unified Parkinson’s Disease Rating Scale (UPDRS III) score (10). Based on disease phenotypes, the gait differences between tremor-dominant (TD) and postural instability and gait difficulty (PIGD) were compared (11).

It is worth noting that gait disorder is not unique in PD (12); it is also very common in the field of cognitive impairment. Ghoraani et al. divided participants into healthy individuals and individuals with mild cognitive impairment and Alzheimer’s disease, and they found that there was a significant positive correlation between the degree of cognitive impairment and the severity of gait disorder (13). Moreover, gait abnormalities are also very common among elderly people, primarily manifesting as slowed walking speed, reduced step length, increased step width, and prolonged double support phase (14), and as age increases, these gait changes became increasingly pronounced.

Among PD patients, the severity of motor symptoms is closely related to the degree of gait disorder (3) and cognitive impairment (15), and cognitive impairment also has a significant correlation with gait disorder (13). There is obviously a complex relationship among motor symptoms, cognitive function, and gait characteristics in PD patients. However, most current studies on the gait of PD patients have limitations. Participants were often limited to those with a Mini-Mental State Examination (MMSE) score of 24 or above (16), or their cognitive status was completely ignored. Although some researchers have noticed the relationship among the three, most of them divided PD patients into a cognitive impairment group and a cognitive normal group and conducted a comparative analysis of the gait differences between the two groups (17). Several studies investigated the associations between cognitive and gait disorders in individuals with PD during single-task and dual-task walking (18, 19) and demonstrated that PD increased reliance on cognition for gait control (20). Based on this finding, some investigations showed that reducing attentional costs (21) could improve gait function (22). However, these studies did not explore the relationships among the three factors in detail.

Mediation analysis is used when the researcher seeks to understand, explain, or test a hypothesis about how or by what process or mechanism a variable X transmits its effect on Y. A mediator variable M is causally located between X and Y and is the conduit through which X transmits its effect on Y. A mediator can be most anything—a psychological state, a cognitive or affective response, or a biological change (23). Some mediation models have been used in PD. Scholl et al. recently showed that cognitive function mediated the relationship between PD severity and freezing-of-gait severity (24). However, they used the freezing-of-gait questionnaire to assess the severity of freezing of gait, but did not involve specific quantitative evaluations of gait parameters such as gait speed and stride length. Another study used a mediation model to relate gait metrics, UPDRS, and outcomes (e.g., falls risk) (25), but they did not consider the impact of cognition. Dubbioso et al. investigated cognitive impairment, gait variability, and fall risk in amyotrophic lateral sclerosis and showed that a mild cognitive impairment is associated with exaggerated gait variability and predicts the occurrence and number of short-term falls, but their study was applied in amyotrophic lateral sclerosis, not in PD (26).

This study aims to conduct an in-depth analysis of the relationships among Movement Disorder Society-Sponsored Revision of the Unified Parkinson’s Disease Rating Scale Part III (MDS-UPDRS III) score (27), the Montreal Cognitive Assessment (MoCA) score (28), and gait parameters by constructing a mediation model. The aim of this study is to reveal the mechanism among motor symptoms, cognitive function, and gait characteristics in PD.

## 2 Methods

### 2.1 Participants

A total of 28 patients with primary PD were included in this study, with 12 men and 16 women. The inclusion criteria were as follows: (1) meeting the diagnostic criteria for primary PD established by the Movement Disorder Society (MDS) in 2015 (29); (2) H&Y stage ranged from 1 to 3; (3) age ≥40 years; and (4) being able to complete the test independently without the assistance of others. The exclusion criteria included the following: (1) Atypical Parkinsonian syndrome; (2) severe systemic and other neurological diseases; and (3) uncorrected visual impairments or diseases that may change gait patterns. This study was approved by the Ethics Committee of Central Hospital of Dalian University of Technology (YN2022-039-57). Each participant signed a consent form before participating in this study.

### 2.2 Clinical assessment

This study collected the demographic and clinical information of the patients, including age, gender, height (cm), weight (kg), disease duration, and levodopa equivalent daily dose (LEDD) (30). All participants underwent a comprehensive neurological examination and clinical scale assessment by experts in movement disorders. MoCA was used to evaluate the cognitive function, and the test was completed during the “ON” state (i.e., the stage when anti-Parkinson drugs exert their full efficacy). The H&Y staging (31) and MDS-UPDRS III scales were used to assess the disease severity, and they were conducted during the “OFF” state (i.e., the stage at least 12 h without using any anti-Parkinson drugs).

### 2.3 Gait assessment

In this study, a self-developed measurement system was used to collect gait data. The structure of the gait data acquisition system was shown in our previous study (32). This system consisted of a handheld control terminal and two sensor nodes (the sampling frequency was 200 Hz). The sensor nodes integrated high-precision sensing and data processing modules, with the following specific configurations: (1) a three-axis accelerometer (dynamic range: ±18 g, sensitivity (/LSB): 0.833 mg, bandwidth (kHz): 330, and alignment error (deg): 0.2); (2) a three-axis gyroscope (dynamic range: ±1,000 deg./s, sensitivity (/LSB): 0.04 deg./s, bandwidth (kHz): 330, and alignment error (deg): 0.05); (3) a microcontroller; (4) a WIFI wireless communication module; and (5) a 450-mAh lithium battery. The sensor nodes were installed on the side of the lateral malleolus of each foot. The handheld terminal supported wireless command transmission and could regulate the working state of the sensors wirelessly. The collected data were uploaded through a wireless network for gait analysis.

The gait parameters collected in this study include plantar dorsiflexion angle (PDA) (left/right, L/R), stride length (L/R), velocity, cadence, stride time, support phase symmetry, and swing phase symmetry (Table 1). The calculation method for gait parameters had been described in a previous study (33). All data was collected during the “OFF” state of the drugs. The participants independently completed walking on a 10-m obstacle-free path at a natural and comfortable speed. Protective facilities were provided throughout the experiment to avoid falling. At the same time, a standardized operation process was adopted, such as avoiding the interference of repetitive verbal cues on the gait pattern, and the data of the first and last steps were excluded in order to ensure the accuracy of the analysis. The accuracy of the gait data acquisition system had been validated in a previous study (34); and compared to the gold standard (optical motion capture), the position estimation error is <1% with regard to three-dimensional motion.

**Table 1:** Specific definitions of gait parameters in this study.

Gait parameters	Definition
Plantar dorsiflexion angle (PDA) (°)	The absolute value of the angle between the foot and the ground at heel-strike moment.
Stride length (m)	Distance between two consecutive heel-strikes, that is, the distance between the landing points of the same feet.
Velocity (m/s)	Calculated by dividing stride length by stride time.
Cadence (steps/min)	Steps per minute.
Stride time (s)	Duration between two heel strikes of the same foot.
Support phase symmetry	$\frac{2*|\text{support phase symmetry}\;(L)-\text{support phase symmetry}\;(R)|}{\text{support phase symmetry}\;(L)+\text{support phase symmetry}\;(R)}$ , the smaller value indicates the better symmetry.
Swing phase symmetry	$\frac{2*|\text{swing phase symmetry}\;(L)-\text{swing phase symmetry}\;(R)|}{\text{swing phase symmetry}\;(L)+\text{swing phase symmetry}\;(R)}$ , the smaller value indicates the better symmetry.

L, left; R, right.

### 2.4 Statistical analysis

This study used SPSS 26.0 (IBM Corporation, Armonk, NY) for statistical analysis. Continuous variables with normal distributions were presented as means ± standard deviations (*x̄* ± *s*). Continuous variables with non-normal distributions were presented as medians and interquartile distances [*M* (*P25*, *P75*)], and the count variables were described by frequency. The correlation between clinical assessment indicators and gait parameters was analyzed using Spearman’s correlation. A *p*-value of < 0.05 was considered statistically significant. The figures were configured using OriginPro 2021 (OriginLab Corporation, Northampton, Massachusetts, USA).

### 2.5 Mediation model

Considering that the MDS-UPDRS III score was correlated with the severity of gait disorder and MoCA score, the MoCA score was also correlated with the severity of gait disorder. This study was conducted to investigate the mediation effect of MoCA between MDS-UPDRS III score and gait parameters. Statistical analysis found that age had no significant correlation with MDS-UPDRS III score, but was associated with MoCA score, suggesting that age is a confounder. Based on this, this study included age as a covariate in the mediation model.

**Figure 1:**
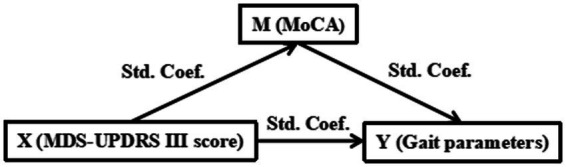
Conceptual diagram of mediation model 4. It illustrating the relationship between X (MDS-UPDRS III score), M (MoCA), and Y (Gait parameters). Arrows indicate direction with “Std. Coef.” labels.

Mediation analysis was performed using the PROCESS macro for SPSS, which employed a regression-based approach to examine the indirect effects; specifically, we conducted a simple mediation analysis following Model 4 according to the Bootstrap method proposed by Hayes (35). This study selected the gait parameters that significantly correlated with the MDS-UPDRS III score, including PDA (L/R), stride length (L/R), velocity, and swing phase symmetry, and included them in the model. Among them, the MDS-UPDRS III score was taken as the independent variable (X), the MoCA score was taken as the mediator variable (M), each gait parameter was taken as the dependent variable (Y), and age was taken as a covariate variable in the mediation model (Figure 1). The significance of the total effect, direct effect, and indirect effect was tested using bootstrapping with 5,000 resamples, and a 95% confidence interval (CI) was reported. The effect was considered statistically significant if the 95%CI did not include 0.

## 3 Results

### 3.1 Demographic and clinical characteristics

A total of 28 PD patients were included in this study, and their demographic and clinical characteristics were collected. Among them, there were 12 men and 16 women, with an average age of 68.71 ± 8.93 years, a height of 164.32 ± 7.72 cm, and a weight of 65.71 ± 9.26 kg, as shown in the first four lines of Table 2. In terms of clinical characteristics, the disease duration ranged from 1 to 8 years, the MoCA score was 21.14 ± 4.31 points, the H&Y stage ranged from stage 1 to stage 3, the MDS-UPDRS III score was 37.57 ± 15.54 points, and the LEDD was between 0 and 750 mg, as shown in lines 5–9 of Table 2. In addition, the gait parameters of PD patients are detailed in lines 10 to 18 of Table 2.

**Table 2:** Demographic and clinical characteristics.

Variables	PD patients (*n* = 28)
Gender (male/female)	12/16
Age (years)	68.71 ± 8.93
Height (cm)	164.32 ± 7.72
Weight (kg)	65.71 ± 9.26
Disease duration (years)	1–8
MoCA (score)	21.14 ± 4.31
H&Y stage	1–3
MDS-UPDRS III (score)	37.57 ± 15.54
LEDD (mg)	0–750
PDA (L) (°)	7.31 (2.23, 12.09)
PDA (R) (°)	8.28 ± 5.60
Stride length (L) (m)	0.97 (0.73, 1.08)
Stride length (R) (m)	0.85 ± 0.28
Velocity (m/s)	0.73 ± 0.27
Cadence (steps/min)	52.04 ± 6.91
Stride time (s)	1.12 (1.05, 1.23)
Support phase symmetry	3.09 (1.64, 4.85)
Swing phase symmetry	3.27 (1.59, 7.84)

PD, Parkinson’s disease; MoCA, Montreal Cognitive Assessment; H&Y, Hoehn and Yahr; MDS-UPDRS III, Movement Disorder Society-Sponsored Revision of the Unified Parkinson’s Disease Rating Scale Part III; LEDD, Levodopa Equivalent Daily Dose; PDA, Plantar dorsiflexion angle; L, Left; R, Right.

### 3.2 Correlation between clinical assessment indicators and gait parameters

**Figure 2:**
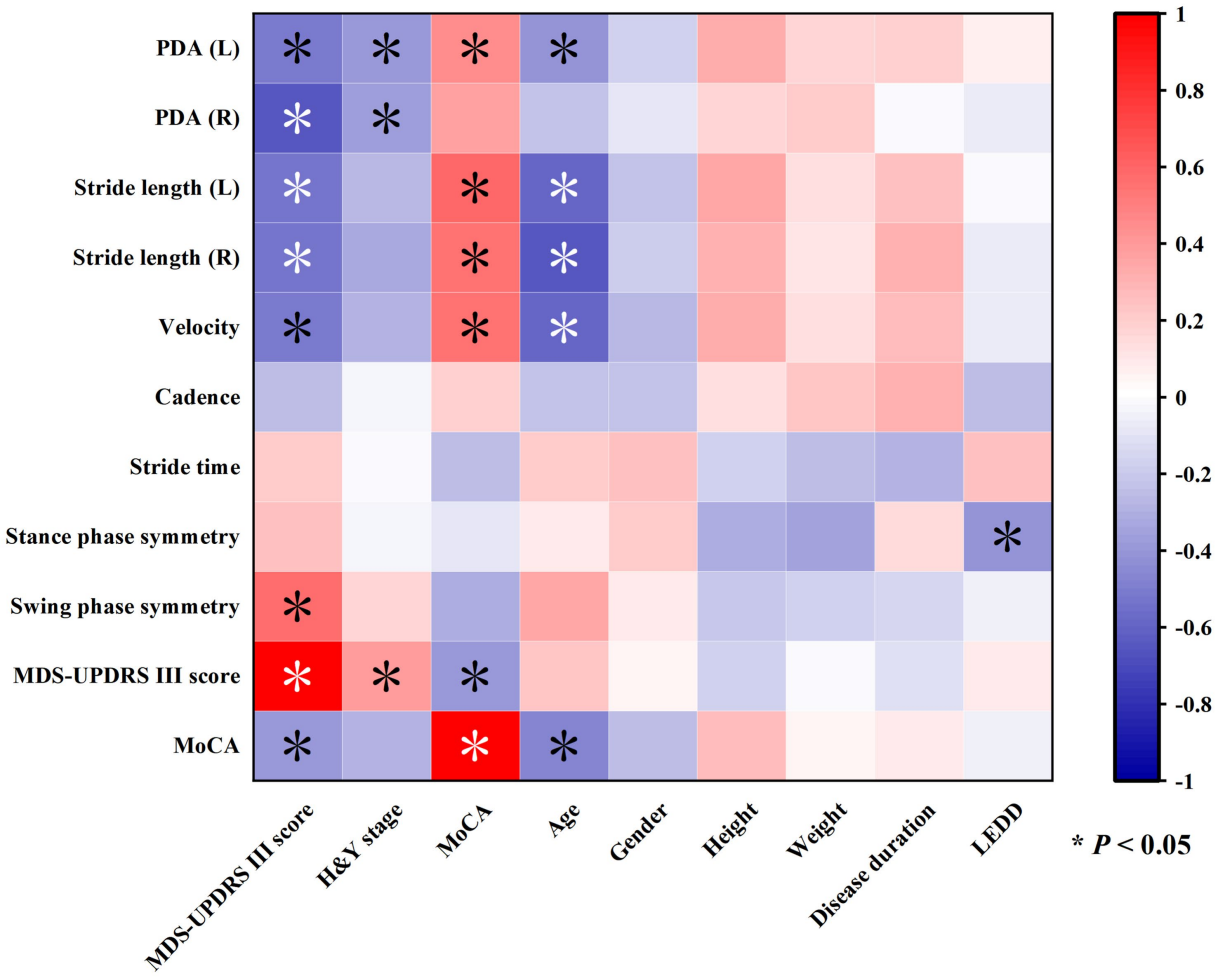
Heatmap showing correlations among various gait and clinical parameters. Colors range from red to blue, indicating positive and negative correlations, respectively. Asterisks mark significant correlations with *p*-values less than 0.05.

This study explored the correlations between clinical assessment indicators and gait parameters, and the results were visually presented through a correlation heatmap (Figure 2). The data showed that the MDS-UPDRS III score was negatively correlated with PDA (L/R), stride length (L/R), and velocity, and positively correlated with swing phase symmetry. There was no correlation between MDS-UPDRS III score and cadence, stride time, and support phase symmetry. MoCA score was positively correlated with PDA (L), stride length (L/R), and velocity, but there was no significant correlation between MoCA score and PDA (R), cadence, stride time, support phase symmetry, and swing phase symmetry. Additionally, age was negatively correlated with PDA (L), stride length (L/R), and velocity but had no correlations with other gait parameters.

Notably, the MDS-UPDRS III score was negatively correlated with the MoCA score but not with age, and the MoCA score was negatively correlated with age.

Further analysis of the relationships between gait parameters and demographic characteristics (gender, height, weight) revealed no significant correlations, suggesting that these factors may have limited influence on gait parameters in this study.

### 3.3 Mediation model

#### 3.3.1 The mediation effect of MoCA in the relationship between MDS-UPDRS III score and PDA (L/R)

**Figure 3:**
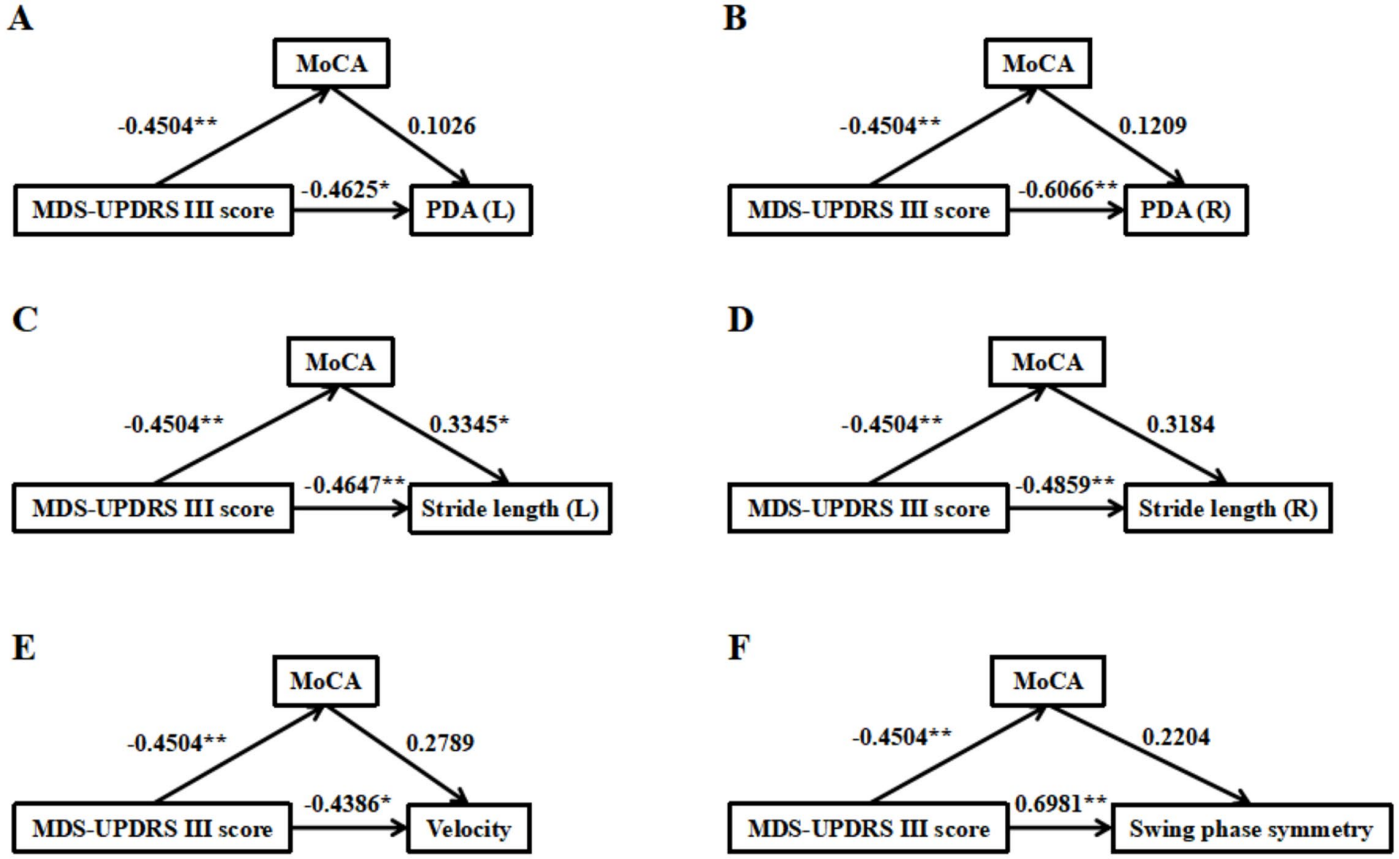
Mediation effect of MoCA in the relationship of MDS-UPDRS III score and gait parameters (labeled **A–F**, including PDA (left, right), stride length (left, right), velocity and swing phase symmetry). Values on arrows indicate correlation strengths, with significance levels marked by asterisks. *p < 0.05, **p < 0.01, ***p < 0.001.

The diagram of the mediation effect of MoCA in the relationship between MDS-UPDRS III score and PDA (L/R) are shown in Figures 3A,B, and the regression analysis among the variables are shown in Table 3, indicating that MDS-UPDRS III score had a negative relationship with PDA (L/R) and MoCA, but MoCA had no relationship with PDA (L/R).

**Table 3:** Regression analysis of variable relationships.

Model	Dependent variable	Independent variable	*R* ^2^	*F*	Coef.	Bootstrap 95% CI		Std. Coef.	*p*-value
						LLCI	ULCI		
MDS-UPDRS III score→ MoCA→PDA (L)	PDA (L)	MDS-UPDRS III score	0.3410	6.4669	−0.2339	−0.3925	−0.0754	−0.5087	**0.0055**
	MoCA	MDS-UPDRS III score	0.4527	10.3403	−0.1249	−0.2120	−0.0378	−0.4504	**0.0068**
	PDA (L)	MDS-UPDRS III score	0.3467	4.2458	−0.2127	−0.4002	−0.0252	−0.4625	**0.0279**
		MoCA			0.1701	−0.5931	0.9333	0.1026	0.6497
MDS-UPDRS III score→ MoCA→PDA (R)	PDA (R)	MDS-UPDRS III score	0.4304	9.4456	−0.2382	−0.3537	−0.1227	−0.6610	**0.0003**
	MoCA	MDS-UPDRS III score	0.4527	10.3403	−0.1249	−0.2120	−0.0378	−0.4504	**0.0068**
	PDA (R)	MDS-UPDRS III score	0.4384	6.2453	−0.2186	−0.3548	−0.0823	−0.6066	**0.0029**
		MoCA			0.1571	−0.3974	0.7116	0.1209	0.5641
MDS-UPDRS III score→ MoCA→ Stride length (L)	Stride length (L)	MDS-UPDRS III score	0.6203	20.4233	−0.0114	−0.0163	−0.0066	−0.6153	**0.0001**
	MoCA	MDS-UPDRS III score	0.4527	10.3403	−0.1249	−0.2120	−0.0378	−0.4504	**0.0068**
	Stride length (L)	MDS-UPDRS III score	0.6816	17.1219	−0.0086	−0.0139	−0.0033	−0.4647	**0.0025**
		MoCA			0.0224	0.0009	0.0439	0.3345	**0.0420**
MDS-UPDRS III score→ MoCA→ Stride length (R)	Stride length (R)	MDS-UPDRS III score	0.6295	21.2416	−0.0114	−0.0161	−0.0067	−0.6293	**< 0.0001**
	MoCA	MDS-UPDRS III score	0.4527	10.3403	−0.1249	−0.2120	−0.0378	−0.4504	**0.0068**
	Stride length (R)	MDS-UPDRS III score	0.6850	17.3994	−0.0088	−0.0139	−0.0037	−0.4859	**0.0017**
		MoCA			0.0208	−0.0001	0.0417	0.3184	0.0508
MDS-UPDRS III score→ MoCA→ Velocity	Velocity	MDS-UPDRS III score	0.5274	13.9488	−0.0098	−0.0149	−0.0047	−0.5643	**0.0005**
	MoCA	MDS-UPDRS III score	0.4527	10.3403	−0.1249	−0.2120	−0.0378	−0.4504	**0.0068**
	Velocity	MDS-UPDRS III score	0.5700	10.6034	−0.0076	−0.0134	−0.0019	−0.4386	**0.0115**
		MoCA			0.0175	−0.0059	0.0409	0.2789	0.1363
MDS-UPDRS III score→ MoCA→ Swing phase symmetry	Swing phase symmetry	MDS-UPDRS III score	0.3374	6.3643	0.2376	0.1004	0.3748	0.5988	**0.0015**
	MoCA	MDS-UPDRS III score	0.4527	10.3403	−0.1249	−0.2120	−0.0378	−0.4504	**0.0068**
	Swing phase symmetry	MDS-UPDRS III score	0.3640	4.5778	0.2770	0.1174	0.4367	0.6981	**0.0015**
		MoCA			0.3154	−0.3345	0.9653	0.2204	0.7923

MDS-UPDRS III, Movement Disorder Society-Sponsored Revision of the Unified Parkinson’s Disease Rating Scale Part III; MoCA, Montreal Cognitive Assessment; PDA, Plantar dorsiflexion angle; L, Left; R, Right; Coef. = Coefficients; Std. Coef. = Standardized coefficients. P value < 0.05 were shown in bold.

As shown in Table 4, the upper and lower limits of the 95% CI of the total effect and direct effect did not include 0, but the upper and lower limits of the 95% CI of the indirect effect included 0. This finding indicated that a higher MDS-UPDRS III score was associated with smaller PDA (direct effect L: 90.94%, direct effect R: 91.77%), and this association was not mediated by MoCA score.

**Table 4:** Decomposition of total effects, direct effects, and indirect effects.

Association	Model effect	Effect size	SE	Bootstrap 95% CI		*P*-value	Effect proportion
				LLCI	ULCI		
MDS-UPDRS III score→MoCA→PDA (L)	Total effect	−0.2339	0.0770	−0.3925	−0.0754	0.0055	100%
	Direct effect	−0.2127	0.0908	−0.4002	−0.0252	0.0279	90.94%
	Indirect effect	−0.0212	0.0473	−0.1249	0.0680	> 0.05	N/A
MDS-UPDRS III score→MoCA→PDA (R)	Total effect	−0.2382	0.0561	−0.3537	−0.1227	0.0003	100%
	Direct effect	−0.2186	0.0660	−0.3548	−0.0823	0.0029	91.77%
	Indirect effect	−0.0196	0.0329	−0.0898	0.0416	> 0.05	N/A
MDS-UPDRS III score→MoCA→Stride length (L)	Total effect	−0.0114	0.0024	−0.0163	−0.0066	0.0001	100%
	Direct effect	−0.0086	0.0026	−0.0139	−0.0033	0.0025	75.44%
	Indirect effect	−0.0028	0.0013	−0.0058	−0.0005	< 0.05	24.56%
MDS-UPDRS III score→MoCA→Stride length (R)	Total effect	−0.0114	0.0023	−0.0161	−0.0067	< 0.0001	100%
	Direct effect	−0.0088	0.0025	−0.0139	−0.0037	0.0017	77.19%
	Indirect effect	−0.0026	0.0013	−0.0054	−0.0003	< 0.05	22.81%
MDS-UPDRS III score→MoCA→Velocity	Total effect	−0.0098	0.0025	−0.0149	−0.0047	0.0005	100%
	Direct effect	−0.0076	0.0028	−0.0134	−0.0019	0.0115	77.55%
	Indirect effect	−0.0022	0.0013	−0.0052	0.0001	> 0.05	N/A
MDS-UPDRS III score→MoCA→Swing phase symmetry	Total effect	0.2376	0.0666	0.1004	0.3748	0.0015	100%
	Direct effect	0.2770	0.0774	0.1174	0.4367	0.0015	116.58%
	Indirect effect	−0.0394	0.0404	−0.1313	0.0262	> 0.05	N/A

MDS-UPDRS III, Movement Disorder Society-Sponsored Revision of the Unified Parkinson’s Disease Rating Scale Part III; MoCA, Montreal Cognitive Assessment; PDA, Plantar dorsiflexion angle; L, Left; R, Right; SE, Standard error; CI, Confidence interval; LLCI, Lower limits of the 95% confidence interval; ULCI, Upper limits of the 95% confidence interval; N/A, not applicable.

#### 3.3.2 The mediation effect of MoCA in the relationship between MDS-UPDRS III score and stride length (L/R)

The diagram of the mediation effect of MoCA in the relationship between MDS-UPDRS III score and stride length (L/R) are shown in Figures 3C,D, and the regression analysis among the variables are shown in Table 3, which indicated that the MDS-UPDRS III score had a negative relationship with stride length (L/R) and MoCA, and MoCA had a positive relationship with stride length (L), but had no relationship with stride length (R) (*p* = 0.0508).

As shown in Table 4, the upper and lower limits of the 95% CI of the total effect, direct effect, and indirect effect did not include 0. This finding indicated that a higher MDS-UPDRS III score was associated with shorter stride length (direct effect L: 75.44%, direct effect R: 77.19%), and this association was partially mediated by the MoCA score (indirect effect L: 24.56%, indirect effect R: 22.81%).

The mediation effect of MoCA in the relationship between MDS-UPDRS III score and stride length (L/R) was significant before and after including age as a covariate variable, suggesting that the confounding effect of age was limited, and the association among MDS-UPDRS III score, MoCA, and stride length (L/R) had strong stability.

#### 3.3.3 The mediation effect of MoCA in the relationship between MDS-UPDRS III score and velocity

The diagram of the mediation effect of MoCA in the relationship between MDS-UPDRS III score and velocity is shown in Figure 3E, and the regression analysis among the variables is shown in Table 3, indicating that MDS-UPDRS III score had a negative relationship with velocity and MoCA, while MoCA had no relationship with velocity.

According to Table 4, the upper and lower limits of the 95% CI of the total effect and direct effect did not include 0, but the upper and lower limits of the 95% CI of the indirect effect included 0. This finding indicated that a higher MDS-UPDRS III score was associated with lower velocity (direct effect: 77.55%), and this association was not mediated by MoCA score.

Interestingly, the mediation effect of MoCA was significant before including age as a covariate variable, but it became non-significant after including age, indicating a notable confounding effect of age. Further comparison of the *R*^2^ values (Table 5) revealed that, when predicting MoCA with MDS-UPDRS III score alone, the *R*^2^ value increased from 0.3006 to 0.4527 (Δ*R*^2^ = 0.1521) after including age, reflecting the additional explanatory power of age on MoCA. When predicting velocity with both MDS-UPDRS III score and MoCA, the *R*^2^ value increased from 0.5318 to 0.5700 (Δ*R*^2^ = 0.0382) after including age, reflecting the additional explanatory power of age on velocity. The larger increase in *R*^2^ for MoCA (0.1521 > 0.0382) suggested that age had a stronger influence on MoCA, and the suppression of the mediation effect was attributed to the confounder of age.

**Table 5:** Comparison of *R*^2^ values before and after including age as a covariate variable for the association among MDS-UPDRS III score, MoCA and velocity.

Dependent variable	Independent variable	*R*^2^ before including age	*R*^2^ after including age	Difference (Δ*R*^2^)
MoCA	MDS-UPDRS III score	0.3006	0.4527	0.1521
Velocity	MDS-UPDRS III score	0.5318	0.5700	0.0382
	MoCA			

MDS-UPDRS III, Movement Disorder Society-Sponsored Revision of the Unified Parkinson’s Disease Rating Scale Part III; MoCA, Montreal Cognitive Assessment.

#### 3.3.4 The mediation effect of MoCA in the relationship between MDS-UPDRS III score and swing phase symmetry

The diagram of the mediation effect of MoCA in the relationship between MDS-UPDRS III score and swing phase symmetry is shown in Figure 3F, and the regression analysis among the variables is shown in Table 3, indicating that MDS-UPDRS III score had a positive relationship with swing phase symmetry and had a negative relationship with MoCA, but MoCA had no relationship with swing phase symmetry.

As shown in Table 4, the upper and lower limits of the 95% CI of the total effect and direct effect did not include 0, but the upper and lower limits of the 95% CI of the indirect effect included 0. This finding indicated that a higher UPDRS III score was associated with worse swing phase symmetry (direct effect: 116.58%), and this association was not mediated by MoCA. Notably, the correlation coefficient was greater than 100%, indicating a suppression effect, but it was not statistically significant.

## 4 Discussion

Gait control is a complex process that involves the integration of multiple systems, such as the motor, perceptual, and cognitive processes (36, 37). Once there is dysfunction in these systems, gait abnormalities emerge. In patients with PD, rigidity symptom limits the range of joint movement, and stride length is closely related to the torque of the knee and ankle joints (38). PDA is closely related to the tibialis anterior muscle (39), so PD patients exhibit shortened stride length and reduced PDA. In addition, bradykinesia symptoms in PD patients can directly lead to a decrease in velocity (40). Moreover, the symptoms of PD are mostly left–right asymmetric (41), further exacerbating the degree of asymmetry of gait. Our research findings were consistent with the above findings.

We found that a higher MDS-UPDRS III score was associated with smaller PDA (L/R) and worse swing phase symmetry, and these associations were not mediated by the MoCA score. A higher MDS-UPDRS III score was associated with shorter stride length, and this association was partially mediated by MoCA score, and the mediation effect had strong stability and was less affected by age. A higher MDS-UPDRS III score was associated with slower velocity, and this association was not mediated by MoCA score. The mediation effect held when the age covariate was not included but failed to hold when age was included.

It has been demonstrated that, as PD progresses, gait and cognition deteriorate (42). Associations between cognitive and gait disorders have been consistently demonstrated in individuals with PD during single- and dual-task walking (18, 19) to compensate for declined gait performance, and individuals increase reliance on cognition to control gait (20). Importantly though, cognitive decline may not be a coincidental impairment that accompanies gait deficits but may rather stem from a pathology linked closely to the mechanisms that affect gait in PD (43).

From the neuroanatomical perspective, cognitive function and gait control share the same neural regulatory basis. The prefrontal cortex not only plays an important role in cognitive function but is also a key component of the motor execution pathway (44, 45). This means that damage to cognitive function may interfere with the prefrontal cortex in task situations. Studies have shown that, when the cognitive resources decreased, the gait patterns showed abnormalities (46).

Functional magnetic resonance imaging (fMRI) is used in studies of motor imagery. Notably, compared with healthy controls, PD patients exhibited increased prefrontal activation during gait imagery tasks (47, 48), and this observation reflected heightened cognitive effort, even during the gait preparation phase (2).

Recently, electroencephalography (EEG) and functional near-infrared spectroscopy (fNIRS) have emerged as promising tools for investigating brain function during walking. These dynamic imaging techniques facilitate a better understanding of neural activation patterns throughout the walking process. In PD, motor abnormalities were associated with reduced power in low-frequency bands. This neural feature was also linked to attention and executive function in cognitive processes. Specifically, changes in *β* activity correlated with freezing of gait, while alterations in *γ* activity were associated with motor execution and gait. Additionally, variations in *θ* activity were related to motor preparation (2). Findings from fNIRS studies further indicated that, compared with healthy controls, PD patients showed increased prefrontal activation even during simple walking tasks. This phenomenon suggested that PD patients required greater engagement of cognitive resources to maintain walking function (49). Kang et al. demonstrated the association between attention, frontal-executive function, and gait in patients with PD (50).

Age constitutes another key factor. With advancing age, the total prefrontal volume decreases, including white matter volume and gray matter volume, and the decline of white matter volume is disproportionately greater than the decline of gray matter volume (51). Age-related alterations in synaptic structure and function of the prefrontal cortex, as well as the remodeling of neuronal networks, exhibited a close correlation with age-dependent cognitive changes (52). Compared with younger adults, older individuals relied on the engagement of broader brain regions during motor control, with the prefrontal cortex being a particularly pivotal area (53). Importantly, these prefrontal regions were intricately linked to both cognitive function and gait regulation. This heightened and overlapping neural demand, coupled with age-related structural and functional declines in the prefrontal cortex, ultimately contributed to the development of age-associated gait disorder and cognitive impairment in older adults.

Collectively, these findings enhanced our understanding of the interplay between cognitive function, gait, age, and neural activity in PD, laying a foundation for future research. Moving forward, integrating larger-sample longitudinal designs, more precise cognitive domain-specific assessment tools, and advanced dynamic neuroimaging techniques could further elucidate the causal relationships and shared mechanisms underlying cognitive function and gait disorder in PD. Such advancements may ultimately inform the development of targeted interventions to mitigate these disabling symptoms and improve the quality of life for PD patients. For example, for patients with abnormal gait parameters identified by wearable devices, rehabilitation therapists could prioritize targeted training (e.g., for PDA and swing phase symmetry, motor symptoms should be the primary consideration; for stride length, motor symptoms and cognition should be targeted; and for velocity, motor symptoms and age should be considered) or adjust the intensity/duration of gait training to match the patients’ cognitive reserve and age, thereby improving the efficiency of rehabilitation.

This study had the following limitations. Our study is a cross-sectional study; although we inferred the relationships among the severity of PD, cognition, and gait based on the characteristics of PD and previous literature, our manuscript lacked longitudinal observational data to directly verify these causal relationships. The sample size of this study was small, with a potential risk of false negatives where “latent associations may not be detected.” The potential residual confounding, such as gender, height, weight, education level, different disease phenotypes (such as TD/PIGD), and freezing of gait status, was not included in the mediation model. This study used the MoCA scale to assess cognitive function but did not distinguish specific cognitive domains. For future studies, it is recommended to include a larger sample size, more variables, and adopt scales that cover detailed cognitive domains, which will help provide more comprehensive information.

## 5 Conclusion

This study explored the relationships between motor symptoms (MDS-UPDRS III), cognitive function (MoCA), and gait parameters in PD patients. Key findings included (1) higher MDS-UPDRS III score correlated with smaller PDA, slower velocity, and worse swing phase symmetry, and the association was not mediated by MoCA; (2) higher MDS-UPDRS III score correlated with shorter stride length, and the association was partially mediated by MoCA; (3) the MDS-UPDRS III → MoCA→stride length association demonstrated strong stability and less influenced by age; and (4) the MDS-UPDRS III → MoCA→velocity association was unstable and could be suppressed by age.

Theoretically, these results support a mediation effect of MoCA on stride length. Practically, they guide targeted interventions: motor-focused training for PDA/velocity/swing phase symmetry abnormalities, combined motor-cognitive training for stride length reduction, and age-aware assessment for velocity issues in PD patients.

Limitations include a small sample size (*n* = 28) and a cross-sectional design. Future research should prioritize large-scale longitudinal studies and domain-specific cognitive tests to validate findings.

## Data Availability

The raw data supporting the conclusions of this article will be made available by the authors, without undue reservation.
